# The LLP risk model: an individual risk prediction model for lung cancer

**DOI:** 10.1038/sj.bjc.6604158

**Published:** 2007-12-18

**Authors:** A Cassidy, J P Myles, M van Tongeren, R D Page, T Liloglou, S W Duffy, J K Field

**Affiliations:** 1 Roy Castle Lung Cancer Research Programme, University of Liverpool Cancer Research Centre, Liverpool, L3 9TA, UK; 2 Cancer Research UK Centre for Epidemiology, Mathematics and Statistics Wolfson Institute of Preventive Medicine, London, EC1M 6BQ, UK; 3 Institute of Occupational Medicine, Research Avenue North, Riccarton, Edinburgh, EH14 4AP, UK; 4 Department of Thoracic Surgery, The Cardiothoracic Centre, Liverpool, L14 3PE, UK

**Keywords:** lung carcinoma, risk prediction, model

## Abstract

Using a model-based approach, we estimated the probability that an individual, with a specified combination of risk factors, would develop lung cancer within a 5-year period.

Data from 579 lung cancer cases and 1157 age- and sex-matched population-based controls were available for this analysis. Significant risk factors were fitted into multivariate conditional logistic regression models. The final multivariate model was combined with age-standardised lung cancer incidence data to calculate absolute risk estimates.

Combinations of lifestyle risk factors were modelled to create risk profiles. For example, a 77-year-old male non-smoker, with a family history of lung cancer (early onset) and occupational exposure to asbestos has an absolute risk of 3.17% (95% CI, 1.67–5.95). Choosing a 2.5% cutoff to trigger increased surveillance, gave a sensitivity of 0.62 and specificity of 0.70, while a 6.0% cutoff gave a sensitivity of 0.34 and specificity of 0.90. A 10-fold cross validation produced an AUC statistic of 0.70, indicating good discrimination.

If independent validation studies confirm these results, the LLP risk models’ application as the first stage in an early detection strategy is a logical evolution in patient care.

In addition, being the most common cancer with over 1.3 million incident cases per year, lung cancer has the highest worldwide rate of cancer mortality (Parkin *et al*, 2005). More than half of all cases are diagnosed at an advanced stage when surgical removal is no longer a viable treatment strategy. As a result, the overall 5-year survival rate is low, but stage-specific survival rates differ substantially by stage at presentation (van Rens *et al*, 2000). This raises the possibility that lung cancer may be an attractive candidate for screening, to detect disease at an early stage when treatment would be more effective. Recent results from the International Early Lung Cancer Action Program would appear to support this argument (I-ELCAP Investigators *et al*, 2006). While the International Early Lung Cancer Action Program results are very encouraging, there are also potential negative consequences of screening, including screen-detected false positives.

Although a mortality benefit from spiral CT has not yet been confirmed in ongoing, large-scale randomised studies, the need to specify a high-risk target population is well accepted, and there has been increasing interest in methods of individual risk prediction for lung cancer. Models have been developed for use within high-risk groups (Bach *et al*, 2003), and for the general population (van Klaveren *et al*, 2002), although the latter tend to rely only on age and smoking. While epidemiological risk factors usually show poor discrimination between those who do and do not develop disease (Wald *et al*, 1999), lung cancer is an exception in that a high proportion of cases are attributable to one risk factor, smoking. However, there is room for further improvement in that many long-term smokers do not develop lung cancer. The predictive accuracy of lung cancer risk models may be further improved by the addition of epidemiological risk factors (Freedman *et al*, 2005; Cassidy *et al*, 2007a, 2007b). For instance, Spitz *et al* (2007) have recently developed a risk prediction model that incorporates smoking history variables, environmental tobacco smoke, family history of cancer, prior respiratory disease and occupational exposures (dust and asbestos).

Here, we report a method to calculate absolute risk of lung cancer over a defined period, based on data from a case–control study of lung cancer in Liverpool, the Liverpool Lung Project (LLP) (Field *et al*, 2005). Our aim was to provide a model (hereafter referred to as the LLP risk model) that would estimate the absolute risk of lung cancer for a given individual. This could be utilised for primary and secondary prevention, possibly to help identify those most likely to benefit from CT screening or as an additional resource for medical decision making. A secondary objective was to include in the final model only variables that are readily available to primary care clinicians when patients present (not necessarily with suspected lung cancer), so that it could be applied in the primary care setting to facilitate the referral of high-risk individuals.

## MATERIALS AND METHODS

To be included in the LLP case–control study, incident cases of histologically or cytologically confirmed lung cancer were between 20 and 80 years of age. Lung cancer included cancer in any of the topographic subcategories of code C34 according to the International Classification of Diseases, 9th Revision. Participants were eligible for inclusion provided they were resident within the Liverpool area and gave informed consent for baseline interviews. Both cases and controls were ineligible for the study if they had a previous cancer within 5-years of interview date (excluding melanoma). Two population controls per case were selected from registers of general practitioners in Liverpool and matched to lung cancer cases by year of birth (±2 years) and gender.

A standardised lifestyle questionnaire was used to collect detailed information on socioeconomic and demographic characteristics, medical history, family history of cancer, history of tobacco consumption and lifetime occupational history. Extensive information about tobacco smoking was elicited for all participants including smoking status, inhalation, type of cigarette smoked, number of cigarettes smoked per day, age at start and end. An ever smoker was defined as someone who had smoked at least 100 cigarettes in their lifetime and a current smoker was defined as a participant who reported smoking 2 years prior to the date of the interview. During the interview, all periods of consumption were defined and counted towards total exposure.

Information on history of cancer among first degree relatives (i.e. parents, brothers and sisters and biological children) was recorded, including age of diagnosis, site of cancer and relation to the participant. All participants were asked if they had ever been told by a physician that they had a prior non-malignant lung disease such as asthma, bronchitis, emphysema, pneumonia or tuberculosis at any age at least 2 years before any diagnosis of lung cancer (or date of interview for controls). Furthermore, if participants had ever been diagnosed with a malignant tumour, the primary site was recorded, and their age at the time of diagnosis.

The methodology to assess occupational exposure was based on that used by Siemiatycki *et al* (1991) and has been decribed previously (Cassidy *et al*, 2007b). Briefly, asbestos exposure was determined firstly by collecting detailed information on each job held using general and specialised questionnaires. Each job was then assessed by an expert, who indicated their confidence in the presence of exposure (categorised as possible, probable or certain). Asbestos exposure was further assessed by frequency of exposure, defined as the percentage of working time exposed (categorised as 1–5, 5–30 or >30%), and intensity of exposure (categorised as low, medium or high). To adhere to the study's secondary objective that only variables readily available to clinicians were included, we characterised asbestos exposure as present if an individual was exposed for at least 1 year during their working life.

The study protocol was approved by the Liverpool Research Ethic Committee and all research participants provided written, informed consent in accordance with the Declaration of Helsinki.

### Statistical analyses

Distributions in demographic variables between cases and controls were evaluated by the *χ*^2^ test. Differences between cases and controls in age and self-reported pack-years were tested using the Student's *t*-test. When the data distribution significantly deviated from normal, the Wilcoxon rank sum test was performed. The risk model was developed using conditional logistic regression. The multivariate model was built up in two phases. First, all statistically significant covariates (*P*<0.05) in univariate analyses were included in a multivariate model, and backward stepwise regression was performed, whereby those factors losing their significance (*P*>0.05) in the multivariate analysis were dropped. Those factors not significant in the univariate analyses were subsequently fitted to the multivariate model, with adjustment for the remaining significant effects, to detect effects, which are only seen when the major risk factors are accounted for. Pairwise interaction tests were conducted between all the risk factors in the final multivariate model to ensure that they did not modify each other's effects. Once the final multivariate model was determined, the logistic model was converted to absolute risk using the method described in the Appendix. The classification power for the model was determined by means of a 10-fold cross-validation procedure and by calculations of area under the curve (AUC) in receiver operating characteristic curve analysis. Statistical analyses were performed using STATA release 9.0 (Stata Corporation, 2005).

## RESULTS

Five hundred and seventy-nine incident cases of lung cancer and 1157 population controls were recruited between 1998 and 2005. Overall, the response rate was 58.3% for cases and 61.5% for controls. Caucasians represented approximately 99% of both the cases and the controls. The majority of lung cancer cases in the study population presented with non small cell lung cancer (83.2%). Table 1 shows the distribution of study-specific risk factors between cases and controls. Men constituted the majority of the cases (61.7%) and, accordingly the controls (61.6%). The proportion of ever smokers was significantly higher in cases (95.3%) compared with controls (71%). Significant differences were observed in a panel of epidemiological risk factors including history of lung cancer in a first degree relative (*P*=0.04), prior diagnosis of pneumonia (*P*=0.001), occupational exposure to asbestos (*P*<0.0001) and prior diagnosis of a malignant tumour (*P*<0.0001).

No significant effect of marital status, education or socioeconomic status was observed on lung cancer risk after adjustment for smoking. There was a significant increase in risk amongst individuals with a prior diagnosis of pneumonia both before (odds ratio (OR)=1.62, 95% confidence interval (CI): 1.21–2.17) and after adjustment for smoking (OR=1.70, 95% CI: 1.21–2.39). Participants with a prior diagnosis of emphysema had a significant increase in risk before adjustment (OR=2.19, 95% CI: 1.25–3.84) but not after (OR=1.78, 95% CI: 0.96–3.30). No effect was present for prior asthma, bronchitis and tuberculosis. Sex-specific analyses indicated that the risk of lung cancer remained significantly elevated for males who had a prior diagnosis of pneumonia (OR=1.92, 95% CI: 1.25–2.95), but not for females (OR=1.30, 95% CI: 0.73–2.29). Women who had a prior diagnosis of emphysema exhibited a significantly increased lung cancer risk (OR=2.72, 95% CI: 1.70–3.70), which was not observed in males (OR=1.30, 95% CI: 0.58–2.94). Physician-diagnosed prior cancer was associated with a significantly increased lung cancer risk (OR=2.18, 95% CI: 1.39–3.42) after adjustment for age, sex and smoking. The majority of reported previous cancers were cancers of the skin, which were associated with a 2.2-fold increased lung cancer risk (95% CI: 1.12–4.26) followed by cancers of the breast (OR=4.81, 95% CI: 1.43–16.15). Although there was a significant trend of increasing risk with numbers of affected relatives, there was no significant effect of family history (any *vs* none) of lung cancer in the study population overall or in late-onset cases, regardless of the age of affected relatives. There was, however, a substantial and statistically significant increase in risk where both the lung cancer case and the affected relative were diagnosed with lung cancer before the age of 60 years (OR=4.89, 95% CI: 1.47–16.25). Significantly elevated odds ratios were also observed in connection with an affected relative diagnosed before age 60 regardless of age-at-onset of the case (OR=2.08, 95% CI: 1.20–3.59) (Cassidy *et al*, 2006). Current smokers (OR=13.15, 95% CI: 8.43–20.50) were at higher risk than ex-smokers (OR=5.72, 95% CI: 3.71–8.82). Fitting total years of smoking duration as a continuous covariate, and in 10- and 20-year intervals revealed a steady increase in lung cancer risk. There was a steady increase in risk with increasing pack-years and average amount smoked, although in neither case was as large as that with smoking duration. A significant dose–response effect was observed for the daily number of cigarettes (*P*<0.0001), smoking duration (*P*<0.0001) and smoking pack-years (*P*<0.0001). No association was found between smoking pipes or cigars and risk of lung cancer. A significant increase in risk was observed for those who reported ever exposure to spousal tobacco smoke (OR=1.44, 95% CI: 1.04–1.98). A significant dose–response effect was observed with duration of exposure (*P*=0.01), with the largest increase in risk in the highest exposed group corresponding to more than 50 years of exposure (OR=2.51, 95% CI: 1.33–4.71). A nonsignificant excess risk was observed for ever exposure to secondhand smoke in the workplace (OR=1.29, 95% CI: 0.97–1.73). However, when analysed by duration of exposure, a dose–response effect was not observed (*P*=0.83). There was no evidence of elevated risk of lung cancer due to exposure to secondhand smoke from social sources. The high lifetime exposure prevalence to asbestos seen in this study is manifest as an overall risk of 1.88 (OR=1.88, 95% CI: 1.36–2.59), which was reduced after adjustment for occupational confounders to 1.51 (95% CI: 1.02–2.04).

The final multivariate logistic regression model is presented in Table 2. Significantly increased risks in the multivariate analysis were observed for family history of lung cancer (with particularly high risk in those with a relative aged under 60 at diagnosis of lung cancer) (*P*=0.01), prior diagnosis of pneumonia (*P*=0.002), prior diagnosis of cancer other than lung (*P*=0.005), occupational exposure to asbestos (*P*<0.001) and duration of smoking (*P*<0.001). We observed significant interaction between a prior diagnosis of pneumonia and prior diagnosis of malignant tumour (*P*=0.04), which lost significance when adjusted for other risk factors in the multivariate model (*P*=0.07). Although various measures of smoking were significant in the univariate analysis, only duration of smoking remained significant in the multivariate model. An association between a prior diagnosis of emphysema and lung cancer also lost significance in the multivariate model.

Using the methods described in the Appendix, the absolute risk of lung cancer within a 5-year period was calculated. The diversity of these estimates can be illustrated by comparing a smoker and nonsmoker of similar age. First, the absolute risk for a man aged 77 with a family history of lung cancer (relative aged under 60 at diagnosis), a history of asbestos exposure and no other risk factors is 3.17% (95% CI, 1.67–5.95). Secondly, a man with the same risk factor profile plus a 45-year smoking history has an absolute risk of 28.68% (95% CI, 15.07–47.67). In these examples, smoking history contributes to an approximate ninefold increase in the 5-year absolute risk of lung cancer.

The LLP risk model estimates also illustrate how a substantial risk can be conferred by risk factors other than smoking. Consider a 67-year-old man who has never smoked, but who has a family history of lung cancer (with the affected relative aged under 60 at diagnosis), a history of asbestos exposure and a prior diagnosis of cancer. His estimated risk in the next 5 years is 3.16%. A man of the same age without these three risk factors would have a 5-year risk of 0.43%. The population or baseline risk in UK males of this age (including those with and without risk factors) as calculated from National Statistics (Office for National Statistics, 2005) is approximately 1.5%. The above and other examples are presented in Table 3.

**Figure 1:**
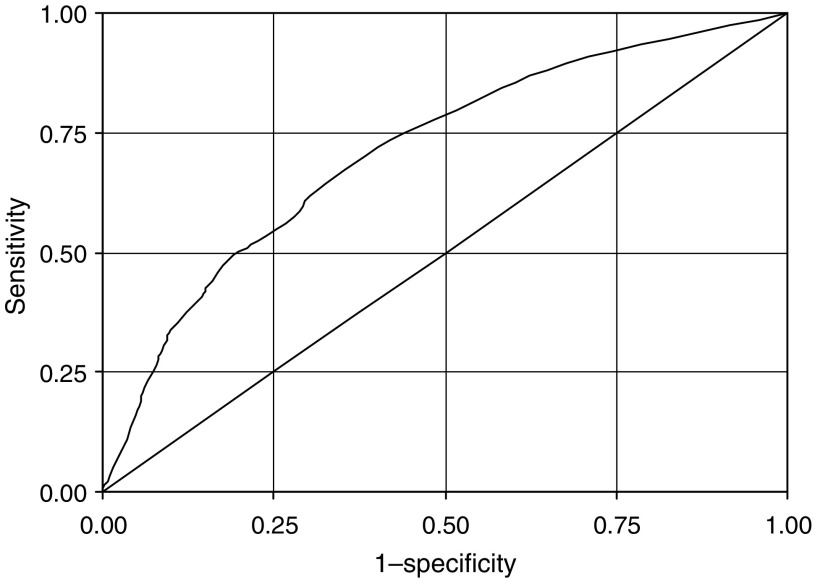
Receiver operating curve for the LLP risk model. The area under the curve is 0.71. The straight line represents the receiver operating characteristic curve expected by chance alone.

Figure 1 shows the receiver operating characteristic curve derived when the model was applied to the case–control population. The area under the curve is 0.71. In addition, a 10-fold cross validation of the LLP risk model produced an area under the curve statistic of 0.70, indicating good discrimination between cases and controls. While this remains to be validated using independent data, the receiver operating characteristic curve gives some insight as to the likely performance of the model using a predefined cutoff. For example, a cutoff at 2.5% would capture 62% of lung cancer cases while including 30% of the controls, giving a sensitivity of 0.62 and specificity of 0.70. A 6% cutoff would capture 34% of lung cancer cases and include only 10% of the controls, giving a sensitivity of 0.34 and a specificity of 0.90.

## DISCUSSION

By combining case–control data with regional incidence rates, we have developed a model to project individual 5-year absolute risks of developing lung cancer. The model has the potential to identify high-risk individuals by focusing on information that can be readily obtained in the primary care setting. The LLP risk model also appears to discriminate between high and low risk, although it will require rigorous validation in separate populations. As well as accounting for the three most important risk factors for lung cancer: age, sex and smoking, the LLP risk model incorporates other important disease risk factors such as family history of lung cancer, occupational exposure to asbestos, prior diagnosis of pneumonia and prior diagnosis of a malignant tumour other than lung cancer.

Similar to previously well-conducted cohort studies (Doll and Peto, 1978; Flanders *et al*, 2003), we have identified the importance of duration of cigarette smoking beyond the absolute amount of tobacco smoked. Indeed, contribution of smoking duration to the model did not change irrespective of whether 10- or 20-year categories were added. Therefore, the broadest category of smoking duration (20 years) was chosen for simplicity. Of the risk factors included in the LLP risk model, a prior diagnosis of malignant tumour is particularly interesting. First, it is important to emphasise that lung cancer cases and controls diagnosed with a malignant tumour (except melanoma) within 5 years of recruitment were excluded from the study. In addition, there were no significant effect modifications between history of previous malignancy and other risk factors in terms of their effect on lung cancer risk. Having had a previous malignancy was associated with a twofold increase in lung cancer risk. This is not unprecedented (Kabat, 1993; Mery *et al*, 2004). Previous studies have reported an increased risk of lung cancer among women receiving radiotherapy for breast cancer (Neugut *et al*, 1993), possibly related to an interaction between radiotherapy and cigarette smoking (Prochazka *et al*, 2002).

Sensitivity and specificity of the LLP risk model compares favourably with previous lung cancer absolute risk models developed by Bach *et al* (2003) and Spitz *et al* (2007). The Bach model is based on a person's age, sex and smoking history, but it is predictive only for individuals between the age of 50 and 75, who smoked 10–60 cigarettes per day for 25–55 years. The Spitz model, like the LLP risk model, expands this concept by incorporating a panel of epidemiological risk factors to more accurately predict an individual's absolute risk of developing lung cancer. One limitation, however, is that cases and controls were frequency matched based on smoking status, perhaps affecting the importance of smoking as a risk factor. We believe that the LLP risk model's simplicity makes it more directly applicable for use in the primary care setting. Indeed, we are about to embark on a feasibility study in a large medical practice, which utilises the LLP risk model as the first stage in an early detection strategy, highlighting the potential importance and clinical relevance of our model.

An obvious strength of this study is that detailed information on the main risk factors such as active smoking, family history of lung cancer and occupational exposure was ascertained by closely supervised, trained interviewers using standardised questionnaires. No proxy interviews were performed. All cases had histologically or cytologically confirmed primary lung tumours. In common with all risk prediction models, the LLP risk model has several limitations. First, the absolute risks estimated for each combination of risk factors are based on relative risks derived from a case–control study. In this study, cases were individuals with newly diagnosed lung cancer identified by surveillance of all the hospitals responsible for the treatment of lung cancer in Liverpool and, after matching for age and sex, controls were selected from population lists that were essentially complete. Thus, cases and controls were both drawn from the same underlying population. However, the refusal rates were high, 42% among cases and 39% among controls. Given that these data did not permit description of smoking patterns of cases and controls refusing to participate, one cannot exclude that the lifestyle characteristics of nonparticipants may differ from the participants leading to an under- or overestimation of the true relative risks. There is also the potential that recall and other information biases could influence our results, as cases and controls were asked to report their lifestyle habits and behaviours for many years prior to interview. For these reasons, it is important that independent validation data be obtained to assess the relative risk features and absolute risk projections from this model. The limitations notwithstanding, this study can inform the debate about the best approach to select individuals at high risk of lung cancer for surveillance or prevention programmes.

Numerous nonrandomised studies have demonstrated that lung cancer can be diagnosed at a significantly earlier stage with CT screening than in current clinical practice (Humphrey *et al*, 2004). Strategies combining smoking history as defined by pack-years have, for the most part, been used as an approach to more efficiently conduct screening in high-risk smoking cohorts (van Klaveren *et al*, 2002), thereby excluding individuals at significantly elevated risk of lung cancer not adequately reflected using these factors. For example, a 60-year-old male with long smoking history would be included while a similar aged never smoker would not. Using the LLP risk model, the 5-year estimated risk for a 60-year-old male with 42 years of smoking and a family history in an affected relative aged 60 years or over is 3.73% (95% CI, 1.85–7.38) while the risk for a 60-year-old male with no smoking history, a family history with an affected relative aged under 60 years, a prior diagnosis of cancer, a prior diagnosis of pneumonia and exposure to asbestos is 3.52% (95% CI, 1.90–6.45). Both these individuals have almost identical risk estimates (a one in 28 and one in 27 chance, respectively of lung cancer in the next 5 years) even though one has never smoked. The LLP risk model potentially provides a means to identify subgroups of both the smoking and nonsmoking populations that may benefit most from prevention or surveillance.

Although some of the absolute risk estimates may seem rather high, they are consistent with population incidence. In the penultimate example in Table 3, a 77-year-old male, never smoker but with an early-onset family history of lung cancer and occupational exposure to asbestos, has an estimated 5-year risk of 3.17%. The general population risk in England and Wales for males aged 75–79 is approximately 2.5%. The modelled risk is slightly higher due to the two risk factors. It is not four times higher as one might anticipate from the odds ratios for early-onset family history and exposure to asbestos, because the general population on average has some exposure to the risk factors in the model. The two most extreme examples in Table 3 have very high-predicted risks of around 30%, mainly but not entirely due to smoking. It should be noted that individuals with this level of risk are very rare; that is not more than 0.7% of cases and 0.3% of controls. This is reflected in the wide area of uncertainty in the confidence intervals, in both examples, showing a range of approximately 15–50% risk.

Although the results suggest that the LLP risk model may be useful for predicting risk, more work is needed to test the applicability of the model in diverse populations, including those from diverse geographic regions. It is clear that some populations, for example, African Americans, have risk factors other than those in our model (Abidoye *et al*, 2007). Marked geographic differences in incidence rates necessitate separate evaluation of the LLP risk model in high- and low-risk areas. Moreover, developing separate models for men and women may allow for the inclusion of distinctive predictors and/or account for their variable distribution, thereby increasing predictive ability.

Although several issues concerning lung cancer risk prediction have been highlighted, we believe that its application as the first stage in an early detection strategy is a logical evolution in patient care. The results presented in this paper suggest that the LLP risk model could predict approximately two-thirds of lung cancer within 5-years, screening only 30% of the population. If resources were limited or the intervention carried such adverse effects as to require a very high-risk population to have a strong benefit–harm balance, a subset of 10% of the population could be identified in which 34% of the cases would arise. The effect of restricting screening to a subpopulation of high-risk individuals will markedly reduce the cost of screening programmes at the expense of missing a proportion of lung cancers in individuals below the cutoff. This ‘high-risk strategy’ aims to help individuals with the greatest need of, and the potential to benefit from early detection. Such stratification on the basis of efficiency implies the difficult decision of where to place the cutoff, but the issue must be addressed nonetheless. It is likely that identifiable genetic susceptibility will, in the future, constitute an important factor in the selection of a more tightly defined risk group. In the meantime, it is appropriate to use a risk prediction model such as ours to identify a high-risk group for CT screening, so long as the results are not used to make inferences about a screening strategy in the general population (Baker *et al*, 2004). If confirmed in validation studies, the LLP risk model could provide individuals and healthcare professionals with an easily obtained estimate of lung cancer risk to guide discussions and decisions regarding prevention and surveillance.

## Figures and Tables

**Table 1:** Distribution of study-specific characteristics of lung cancer cases and healthy controls

	**Cases**		**Controls**		
**Variable**	**No.**	**%**	**No.**	**%**	*P*-value*
*Gender*					
Male	357	61.7	713	61.6	0.99
Female	222	38.3	444	38.4	
					
*Mean age (years; mean±s.d.^a^)*	66.4±9.1		66.3±9.0		0.93
					
*Smoking duration*					
Never	27	4.7	335	29.0	<0.0001
1–19 years	43	7.4	236	20.4	
20–39 years	157	27.1	337	29.1	
40–59 years	321	55.4	234	20.2	
⩾60 years	31	5.4	15	1.3	
					
*Prior diagnosis of pneumonia* b					
No	361	62.3	989	85.5	0.001
Yes	104	18.0	168	14.5	
					
*Occupational exposure to asbestos*					
No	287	64.9	664	76.3	<0.0001
Yes	155	35.1	206	23.7	
					
*Prior diagnosis of malignant tumour*					
No	509	87.6	1091	94.3	<0.0001
Yes	72	12.4	66	5.7	
					
*Family history of lung cancer*					
No	456	78.8	947	82.0	0.04
Early onset (<60 years)	46	7.9	62	5.4	
Late onset (⩾60 years)	77	13.3	148	12.8	

^*^P values were derived from univariate conditional logistic regression.

a s.d.

b Numbers do not add up to total due to missing data.

**Table 2:** LLP multivariate risk model, with unadjusted and adjusted odds ratios and 95% confidence intervals corresponding to the model coefficients

**Risk factor/category**	**Odds ratio^a^**	**(95% CI)**	**Odds ratio^b^**	**(95% CI)**	***P*-value**	**Model coefficient**
*Smoking duration*					<0.001	
Never	1.00	Reference	1.00	Reference		0.000
1–20 years	2.48	(1.47–4.17)	2.16	(1.21–3.85)		0.769
21–40 years	5.81	(3.68–9.18)	4.27	(2.62–6.94)		1.452
41–60 years	19.24	(12.07–30.67)	12.27	(7.41–20.30)		2.507
>60 years	41.74	(17.86–97.56)	15.25	(5.71–40.65)		2.724
*Prior diagnosis of pneumonia*					0.002	
No	1.00	Reference	1.00	Reference		0.000
Yes	1.62	(1.21–2.17)	1.83	(1.26–2.64)		0.602
*Occupational exposure to asbestos*				<0.001		
No	1.00	Reference	1.00	Reference		0.000
Yes	1.94	(1.46–2.59)	1.89	(1.35–2.62)		0.634
*Prior diagnosis of malignant tumour*					0.005	
No	1.00	Reference	1.00	Reference		0.000
Yes	2.55	(1.76–3.71)	1.96	(1.22–3.14)		0.675
*Family history of lung cancer*					0.01	
No	1.00	Reference	1.00	Reference		0.000
Early-onset (<60 years)	1.54	(1.03–2.29)	2.02	(1.18–3.45)		0.703
Late-onset (⩾60 years)	1.08	(0.80–1.46)	1.18	(0.79–1.76)		0.168

a Odds ratios derived from univariate conditional logistic regression.

b Odds ratios derived from multivariate conditional logistic regression.

**Table 3:** Projected 5-year absolute risks and 95% confidence intervals for combinations of risk factors

				**Prior diagnosis of**				
**Gender**	**Age**	**Smoking duration (Years)**	**Family history of lung cancer^a^**	**Malignancy**	**Pneumonia**	**Asbestos exposure**	**Absolute risk (%)**	**(95% CI)**
Female	65	37	Late-Onset	—	Yes	—	2.37	(1.14–4.86)
	68	26	—	—	—	—	1.50	(0.91–2.46)
	69	50	—	—	—	—	4.60	(2.76–7.54)
								
Male	64	42	Late-Onset	Yes	—	—	9.53	(4.52–18.97)
	66	53	—	—	—	Yes	8.75	(4.89–15.18)
	66	48	—	—	Yes	Yes	14.91	(7.70–26.89)
	67	0	Early-Onset	Yes	—	Yes	3.16	(1.42–6.85)
	73	59	Late-Onset	Yes	—	Yes	27.09	(13.57–46.78)
	77	0	Early-Onset	—	—	Yes	3.17	(1.67–5.95)
	77	45	Early-Onset	—	—	Yes	28.68	(15.07–47.67)

a Early onset=<60 years at diagnosis; Late onset =⩾60 years at diagnosis.

**Table A1:** Age- and sex-specific lung cancer incidence rates and estimated α-values relating to 5-year absolute risk

	**Male**		**Female**	
**Age group**	**Incidence rate^a^**	***α*-value**	**Incidence rate^a^**	***α*-value**
40–44	15.5	−9.06	5.97	−9.90
45–49	37.87	−8.16	37.34	−8.06
50–54	88.65	−7.31	68.14	−7.46
55–59	172.26	−6.63	175.24	−6.50
60–64	329.02	−5.97	230.6	−6.22
65–69	487.42	−5.56	288.06	−5.99
70–74	616.45	−5.31	464.99	−5.49
75–79	950.61	−4.83	594.19	−5.23
80–84	1096.42	−4.68	497.09	−5.42

a Lung cancer incidence rate per 100 000 person-years, Liverpool, 2002–2004.

**Table A2:** Covariance matrix of logistic regression estimates

					**Family history of lung cancer**		**Smoking duration (years)**			
		**Prior pneumonia**	**Asbestos exposure**	**Prior cancer**	**<60 years**	**⩾60 years**	**1–20**	**21–40**	**41–60**	**>60**
Prior pneumonia		0.036								
Asbestos exposure		0.001	0.029							
Prior cancer		0.002	0.000	0.058						
Family history	<60 years	0.004	0.002	0.001	0.075					
Family history	⩾60 years	−0.001	−0.001	−0.002	0.007	0.042				
Smoking duration	1–20 years	0.000	−0.001	0.002	−0.004	−0.002	0.085			
Smoking duration	21–40 years	−0.001	0.000	0.001	0.000	−0.001	0.048	0.062		
Smoking duration	41–60 years	0.002	0.001	0.001	−0.003	−0.002	0.051	0.049	0.066	
Smoking duration	⩾60 years	0.006	0.001	0.000	−0.002	−0.007	0.051	0.048	0.058	0.25

## Appendix

### Conversion of multivariate logistic model to absolute risk estimates

Estimates of 5-year lung cancer absolute risk were developed using methods similar to those of Gail *et al* (1989) and Chen *et al* (2006), combining relative risk models with local rates for lung cancer incidence. Age-standardised lung cancer incidence rates were obtained from the North West Cancer Intelligence Service for the period 1 January 2002 through 31 December 2004.

The logistic regression model arrived at after multivariate analysis is of the form

where *x*_*i*_ are the risk factor values, *β*_*i*_ the log odds ratios and *p* is the probability of disease. The above can be re-expressed after simple algebra as

It is not possible to estimate the *α* using case–control data. Assuming that the controls are representative of the Liverpool population conditional on the matching variables, age and sex, one can estimate α using age- and sex-specific lung cancer incidence rates for the Liverpool area. Table A1 shows these rates per 100 000 person-years observed in 2002–2004. For example, to estimate the α corresponding to a five-year probability of developing lung cancer for males aged 50–54, one calculates the *α* for which the average value of *p* in equation (A2) over all male controls aged 50–54 is equal to 5 × 0.0008865=0.0044325. Estimation by repeated bisection gives an *α* of −7.307. Table A1 also shows the derived *α*'s and their standard errors, by age group and sex.

One further step is required to calculate an individual's *α*. For a male aged 51 completed years, for example, we assume that his exact age is 51.5. He will therefore spend the next 3.5 years in the age group 50–54 and the subsequent 1.5 years in the age group 55–59. One can therefore calculate his individual *α* as

More generally, for someone aged x+y years, where x is a multiple of 5 and y is 0, 1, 2, 3 or 4, the individual α is calculated as

Thus, for a 51-year-old male with 25 years of smoking and no other risk factors, the 5-year absolute risk of lung cancer is estimated as

In other words, this man has a 0.3% probability of developing lung cancer in the next 5 years.

In addition to providing an estimate of absolute risk, it is helpful to present an estimate of the uncertainty associated with that risk. The variance of an individual logistic regression equation is

where *V*(*β*_i_) and cov(*β*_i_,*β*_j_) are from the estimated variance-covariance matrix of the conditional logistic regression estimates. One assumes that *α* is uncorrelated with the *β*'s. This is conservative, since from Equation (A2), covariances of *α* with the *β*'s will tend to be negative. It can be shown that the variance of *α* is approximately equal to the inverse of the number of cases in the population age-sex stratum used to estimate the incidence, and corresponding to equation (A3)

We use the variance estimate in (A4) to calculate the 95% confidence interval on LR and then transform the endpoints of the confidence interval to the absolute probability scale as in Equation (A2).

To compute the 95% confidence interval, we shall extend the example of a 51-year-old male with 25 years of smoking and no other risk factors. As demonstrated previously, he has a 0.3% risk of developing lung cancer in the next 5 years. From Table A1, the variance of the *α* (intercept) is

The covariance matrix of the logistic regression estimates are presented in Table A2. The variance of the logistic regression equation is 0.062. Thus, the 95% confidence interval on the logistic regression equation is

This gives a confidence interval of (−6.202, −5.107). The 95% confidence interval on the 5-year risk estimate is

That is, on the percentage risk of 0.3%, the 95% confidence interval is 0.2–0.6%, otherwise expressed as absolute risk=0.3 (95% CI, 0.2–0.6).

A C program has been developed that computes the estimates of absolute risk and the corresponding confidence intervals for any combination of risk factors. This will be made available to researchers for further testing of this method.
